# Honokiol Induces Ferroptosis by Upregulating HMOX1 in Acute Myeloid Leukemia Cells

**DOI:** 10.3389/fphar.2022.897791

**Published:** 2022-05-11

**Authors:** Xingrong Lai, Yanhua Sun, Xuedi Zhang, Dan Wang, Jialing Wang, Haihua Wang, Yao Zhao, Xinling Liu, Xin Xu, Haoran Song, Wenjia Ping, Yanli Sun, Zhenbo Hu

**Affiliations:** ^1^ Laboratory for Stem Cell and Regenerative Medicine, Affiliated Hospital of Weifang Medical University, Weifang, China; ^2^ Weifang Medical University, Weifang, China; ^3^ Department of Hematology, Weifang People’s Hospital, Weifang, China; ^4^ Department of Hematology, Affiliated Hospital of Weifang Medical University, Weifang, China; ^5^ School of Life Science and Technology, Weifang Medical University, Weifang, China; ^6^ Department of Laboratory Medicine, Weifang Medical University, Weifang, China

**Keywords:** honokiol, AML, ferroptosis, heme oxygenase (HO)-1, lipid peroxidation

## Abstract

Acute myeloid leukemia (AML) is one of the malignant hematological cancers with high mortality. Finding a more effective and readily available treatment is of the utmost importance. Here, we aimed to identify the anti-leukemia effect of a natural small molecule compound honokiol on a panel of AML cell lines, including THP-1, U-937, and SKM-1, and explored honokiol’s potential biological pathways and mechanisms. The results showed that honokiol decreased the viability of the targeted AML cells, induced their cell cycle arrest at G0/G1 phase, and inhibited their colony-formation capacity. Honokiol also triggers a noncanonical ferroptosis pathway in THP-1 and U-937 cells by upregulating the level of intracellular lipid peroxide and HMOX1 significantly. Subsequent studies verified that HMOX1 was a critical target in honokiol-induced ferroptosis. These results reveal that honokiol is an effective anti-leukemia agent in AML cell lines and may be a potential ferroptosis activator in AML.

## Introduction

Acute myeloid leukemia (AML) with complex karyotypes accounts for 10–14% of all AML patients, and the overall survival rate is less than 20% (Daneshbod et al., 2019; Mrózek et al., 2019). Until now, most AML patients have been treated by chemotherapeutic regimens. While chemotherapy kills leukemia cells, it often results in serious side effects to the patients and drug resistance of the leukemia cells. Although hematopoietic stem cell transplantation can alleviate or even cure AML with complex karyotype, its limited source restricts its wide application in clinical settings (Fenwarth et al., 2021). Therefore, finding a more effective and readily available treatment for AML is of the utmost importance.

Several components with a deep history in traditional Chinese medicine have been proven effective in treating AML. For example, arsenic trioxide combined with all-trans retinoic acid can achieve a complete response in 90–100% of acute promyelocytic leukemia (APL) patients, with an overall survival rate of 86–97% (Lo-Coco et al., 2013; Ni et al., 2019). In addition, Homoharringtonine (HHT) can be a reliable choice for patients with AML, especially for newly diagnosed or patients younger than 60. Additionally, the use of HHT can reduce relapse in elderly intolerant patients (Mi et al., 2021).

Several previous studies proved that lipid metabolism was reprogrammed and lipid synthesis was significantly up-regulated in hematological malignancy (Stuani et al., 2018; Humbert et al., 2021; Ito et al., 2021; Tcheng et al., 2021). Therefore, it may be an effective strategy to treat AML through interfering fatty acid and lipid metabolism.

**FIGURE 1:**
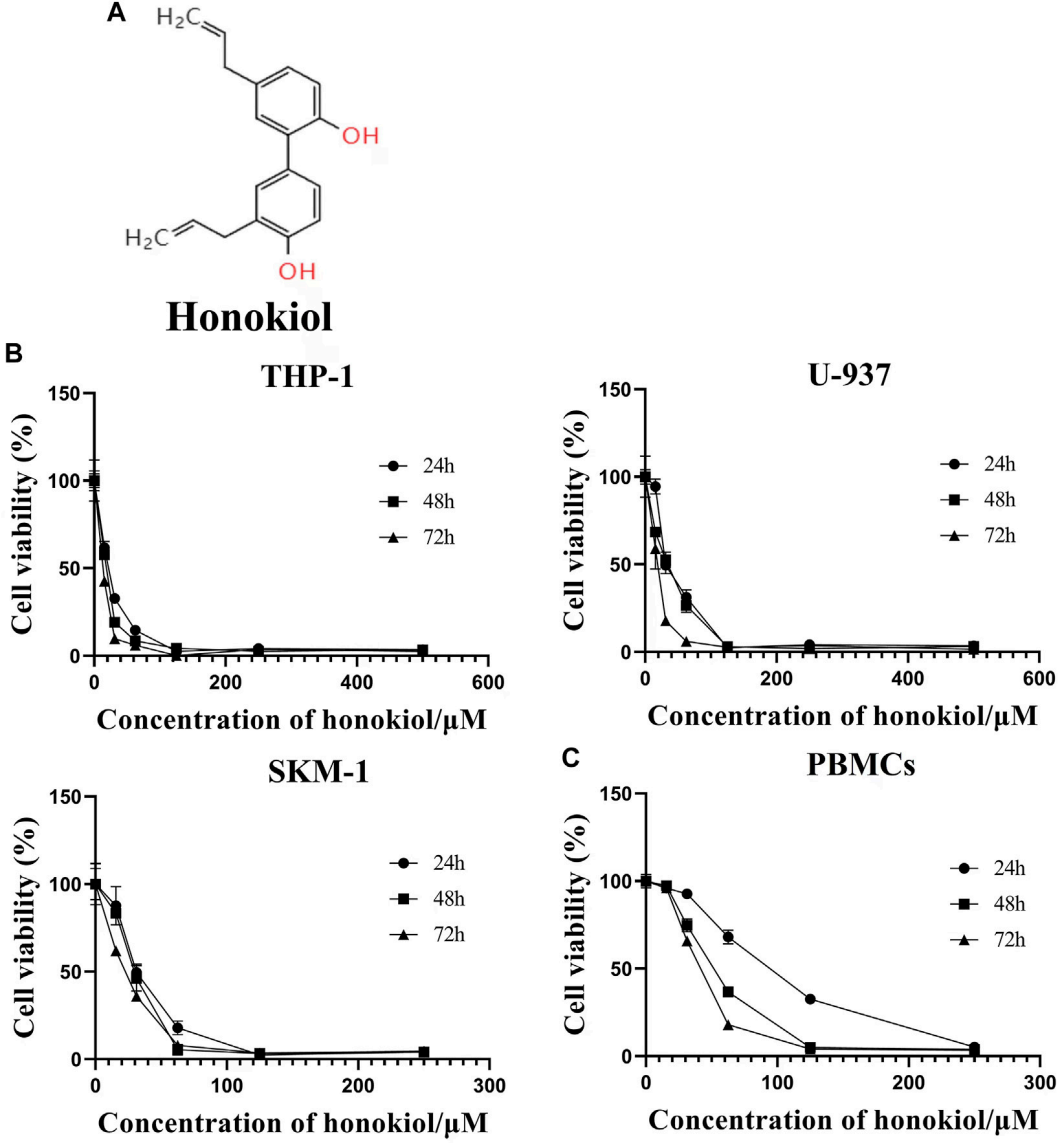
Effects of honokiol on the viability and apoptosis of THP-1, U-937, and SKM-1 cells. **(A)** Chemical structure of honokiol. **(B)** Effects of honokiol on the viability of THP-1, U-937, and SKM-1 cells. Cells were incubated with different concentrations of honokiol for 24, 48, 72 h, then CCK-8 was added, and the absorbance at 450 nm was determined. The points represent mean ± SEM from three independent experiments. **(C)** Cytotoxic effect of honokiol on human PBMCs. PBMCs were incubated with different concentrations of honokiol for 24, 48, 72 h, then CCK-8 reagent was added, and the absorbance at 450 nm was determined. The points represent mean ± SEM from three independent experiments.

Honokiol (Figure 1A), from the mark of the magnolia (Fang et al., 2015), is a biphenolic phytochemical that possesses potent antioxidant, anti-inflammatory, antiangiogenic, antimicrobial, and anti-cancer activities (Banik et al., 2019). In addition, it displayed diverse anti-cancer efficacy in different tumors, including but not limited to prostate cancer, gastric cancer, oral cancer, glioblastoma, skin cancer, ovarian cancer, osteosarcoma, lung cancer, leukemia, liver cancer, and colon cancer (Arora et al., 2012).

Till now, a variety of signaling pathways have been studied to elucidate the anti-cancer effect of honokiol, including apoptosis (Li et al., 2015), EGFR signaling pathway (Leeman-Neill et al., 2010), STAT3 activation cascade pathway (Rajendran et al., 2012), mTOR pathway (Liu et al., 2008), NF-κB signaling pathway (Dikalov et al., 2008), and autophagy pathway (Muniraj et al., 2020). Moreover, honokiol has been proven to play a vital role in modulating fatty acid oxidation in normal cells (Zhu et al., 2018; Li et al., 2020a), whether this mechanism is involved in its anti-leukemia effect has not been determined yet. Furthermore, no study has yet to determine if honokiol can induce ferroptosis in leukemia cells.

Ferroptosis, discovered in 2012 (Dixon et al., 2012), is a ROS-dependent form of lipid peroxidation-induced cell death, accompanied by iron accumulation (Tang et al., 2021a). Generally, there are canonical and noncanonical mechanisms in ferroptosis: the former is induced by dropping the protein level and activity of glutathione peroxidase 4 (GPX4), while the latter is induced by increasing the expression and activation of heme oxygenase 1 (HMOX1) (Tang et al., 2021a).

This study aimed to evaluate the anti-leukemia effect and mechanism of honokiol in the AML cell lines with complex karyotypes. We found that honokiol can induce ferroptosis of AML cells with complex karyotypes by upregulating the expression of HMOX1.

## Materials and Methods

### Chemicals and Equipment

Honokiol purchased from MedChemExpress was dissolved in DMSO and stored at −20°C. RPMI-1640 medium, FBS, penicillin 5,000 U/ml, streptomycin 5,000 mg/ml were purchased from GIBCO (GIBCO,United States). The Propidium iodide (PI)/RNase buffer and the Fluorescein Isothiocyanate (FITC) Annexin V apoptosis detection kit were purchased from BD Biosciences (San Jose, CA, United States). HMOX1 inhibitor ZnPP (Zinc Protoporphyrin Zn(II)-protoporphyrin IX) and ferroptosis inhibitor Ferrostatin -1 were purchased from Sigma-Aldrich LLC (Shanghai, China). SLC7A11/xCT (AB175186), HO-1 (AB189491), and GPX4 were purchased from Abcam (Cambridge, MA, United States). PrimerScript RT kit and SYBR Premix Ex Taq Kit were purchased from TAKARA Bio(Otsu, Japan). Flow cytometry analysis was performed using the CytoFLEX V0-B3-R2 Flow Cytometer (Beckman Coulter, United States). PCR amplification was performed using the Applied Bio 7,500 Fast Real-Time PCR System (Thermo Fisher Scientific, Waltham, MA, United States).

### Cell Lines and Cell Culture

AML cell lines THP-1, U-937, and SKM-1, all monocytes origin with complex karyotypes, obtained from DSMZ (DSMZ, Brunschweig, Germany, https://www.dsmz.de/) were cultured in RPMI 1640 medium containing 10% or 20% FBS and 1% penicillin/streptomycin at 37°C and 5% CO_2_.

### Cell Viability Assay

CCK-8 assay was used to detect cell viability. THP-1, U-937, and SKM-1 cells were seeded into 96-well plates with 6 × 10^3^ cells per well. Simultaneously, the cytotoxicity of honokiol for the peripheral blood mononuclear cells (PBMCs) was also evaluated. Six hours later, the specified compounds were added and cultured for 24, 48, 72 h. Four hours before ending the incubation, 10 μl of CCK-8 was added to each well and incubated for 4 h. The total volume in each well was 100 μl. The absorbance was measured at 450 nm using a microplate reader (Thermo Fisher Scientific, United States).

### Cell-Cycle Analysis

THP-1, U-937, and SKM-1 cells in their logarithmic growth phases were added to a 24-well plate with 2 × 10^5^/well and incubated with different concentrations of honokiol for 24 h before cells were collected. The cells were fixed using 70% pre-cold ethanol overnight and then stained with 400 μl PI (50 μg/ml) and 100 μl RNase A (100 μg/ml) at room temperature for 15 min in the dark. Flow cytometry analysis was performed using CytoFLEX V0-B3-R2 Flow Cytometer (Beckman Coulter, United States) to determine the percentage of cells at every phase of the cell cycle.

### Cell Apoptosis Assay

THP-1, U-937, and SKM-1 cells were incubated with different concentrations of honokiol, respectively. After 24 h, the cells were collected, washed twice using the binding buffer, and incubated with FITC-labeled Annexin-V and PI (BD Biosciences) at room temperature in the dark for 30 min. Cell apoptosis was determined by flow cytometry.

### Colony Formation Assay

5 × 10^3^ THP-1, U-937, and SKM-1 cells were mixed with the indicated concentration of honokiol in 500 μL methylcellulose medium (MethoCult™ H4100, Stemcell Technologies) containing 10% fetal bovine serum, 10% 5,637 cell cultural supernatant, and 1% penicillin/streptomycin, then added to 24-well plates, 500 μl/well. After 14 days of incubation, the total number of cell colonies consisting of at least 50 cells was counted using an inverted microscope. To determine any toxicity of honokiol towards normal cells, normal hematopoietic stem/progenitor cells were isolated and enhanced from cord blood of health donors with informed consent by gradient centrifugation. 1 × 10^3^ enhanced hematopoietic stem/progenitor cells were incubated with specified concentration of honokiol (25, 35 and 45 μM) in 100 µl of methyl-cellulose medium per well of 96-well plate (MethoCult H4434, STEMCELL Technologies, Victoria, Canada) containing cytokines and 10% FBS. The groups without honokiol treatment and with DMSO were used as controls. After about 14 days, the total colony formation numbers of containing all the BFU-E-, CFU-GM-, and CFU-GEMM- derived colonies in different groups were counted under an inverted microscope.

### Detection of Lipid Peroxidation

The liperfluo (Dojindo) was used to detect lipid peroxidation per the manufacturer’s protocol. THP-1, U-937, and SKM-1 cells (5 × 10^5^ cells/well) were seeded in 24-well plates. Six hours later, different concentrations of honokiol were added to these cells. After 12 h incubation, cells were washed with HBSS twice and incubated with 1 µM liperfluo reagent at 37°C, 5% CO_2_ for 30 min. Following that, the cells were washed with HBSS twice. The content of intracellular lipid peroxide was detected by flow cytometry (Beckman Coulter, United States).

### mRNA Sequencing

mRNA sequencing was performed on THP-1 and U-937 cell lines (Hangzhou Lianchuan Biotechnology Co., LTD., Zhejiang, China). After incubation with honokiol for 24 h, the cells were collected, and RNA was extracted from the cell samples. The quality of extracted RNA was evaluated. Total 2 μg RNA was purified and used to construct the cDNA libraries. Sequencing was performed using Illumina HiSeq 4,000 platform. The gene expressions were calculated using fragments per kilobase of exon per million fragments mapped (FPKM) values. False discovery rate (FDR) was used to identify the *p*-value threshold and analyze the differences’ significance. Significantly differentially expressed genes (DEGs) were set with a standard [the adjusted *p*-value < 0.05 and the absolute value of log2 FC.

(fold change) > 1]. Enrichment analysis of the KEGG pathway of DEGs was performed using R language. The enriched KEGG pathway was statistically significant, with the adjusted *p*-value < 0.05.

### Real-Time Quantitative PCR

THP-1, U-937, and SKM-1 cells were incubated with a specific concentration of honokiol. After a certain interval (6 and 12 h), the cells were collected, and total RNA was extracted from the TRIzol samples. The cDNA was reversely transcribed from 1 μg total RNA. Based on the SYBR Green method, HMOX1, SLC7A11, and GPX4 were quantified by RT-qPCR using Applied Biosystems 7,500 Fast Real-Time PCR system. The relative quantification results of gene expression were calculated using the 2^-△△Ct^ method. The primers used are as follows: GAPDH-Forward: 5′-TGG​GTG​TGA​ACC​ATG​AGA​AGT-3′ and GAPDH-Reverse: 5′-TGA​GTC​CTT​CCA​CGA​TAC​CAA-3'; HMOX1-Forward: 5′-AGT​TCA​AGC​AGC​TCT​ACC​GC-3′ and HMOX1-Reverse: 5′-GCA​ACT​CCT​CAA​AGA​GCT​GGA​T-3'; SLC7A11-Forward: 5′-TCT​CCA​AAG​GAG​GTT​ACC​TGC-3′ and SLC7A11-Reverse: 5′-AGA​CTC​CCC​TCA​GTA​AAG​TGA​C-3'; GPX4-Forward: 5′-GAG​GCA​AGA​CCG​AAG​TAA​ACT​AC-3′ and GPX4- Reverse: 5′-CCG​AAC​TGG​TTA​CAC​GGG​AA-3'.

### Western Blotting

Equal amounts (about 40 μg) of cell lysates from samples were loaded to the SDS-PAGE gel, and electrophoresis was performed. After electrophoresis, the proteins were transferred from 12% SDS-PAGE gels onto 0.45 μM PVDF (polyvinylidene fluoride) membranes. First, they were blocked by 5% skim milk in TBST (Tris-buffered saline with 0.1% Tween-20). Then, they were incubated with GAPDH, HMOX1, SLC7A11, or GPX4 antibodies overnight. After that, the membranes were immersed in the HRP-labeled goat anti-rabbit antibody solution at 37°C for 1 h, followed by enhanced chemiluminescence reagent added. The specific protein bands were visualized using the Amersham Imager 600 (GE Healthcare Biosciences, Pittsburgh, PA, United States).

### Statistical Analysis

All experiments were repeated at least three times, and the data were presented as the mean ± SEM. ANOVA analysis or *t*-test was used to determine the control and experimental groups’ statistical significance. Data were considered statistically significant when *p* < 0.05.

## Results

### Honokiol can Effectively Inhibit the Proliferation of Acute Myeloid Leukemia Cells

In order to evaluate if Honokiol can effectively inhibit the proliferation of acute myeloid leukemia cell, we used CCK-8 to detect the effects of honokiol on the viability of AML cells. As shown in Figure 1B, honokiol significantly reduced the cell viability of the three cell lines THP-1, U-937 and SKM-1. According to IC50 shown in Table 1, THP-1 cells were more sensitive than U-937 and SKM-1 cells to honokiol. As the incubation dose and time increased, IC50 of honokiol decreased, indicating the inhibition effect of honokiol on leukemia cells is dependent on dose and time in each cell line. However, no obvious cytotoxic effect was observed for the PBMCs treated with the dose of less than 50 μM of honokiol as shown in Figure 1C.

**TABLE 1:** IC50 Values of Honokiol on AML cell lines.

Cell lines	IC50 (μM) ± SD		
	24 h	48 h	72 h
THP-1	20.64 ± 2.94	17.64 ± 2.51	13.78 ± 0.86
U-937	36.49 ± 2.36	29.72 ± 2.05	17.85 ± 3.23
SKM-1	32.05 ± 1.3	28.71 ± 1.87	21.07 ± 2.86

IC50 values are calculated from cell proliferation assays. IC50 (μM) ± SD: the compound concentration at which cell survival was inhibited by 50% (means ± SD). Values are representative of three independent experiments, and each experiment was performed in triplicate.

### Honokiol Induced the Apoptosis of THP-1, U-937, and SKM-1 Cells

**FIGURE 2:**
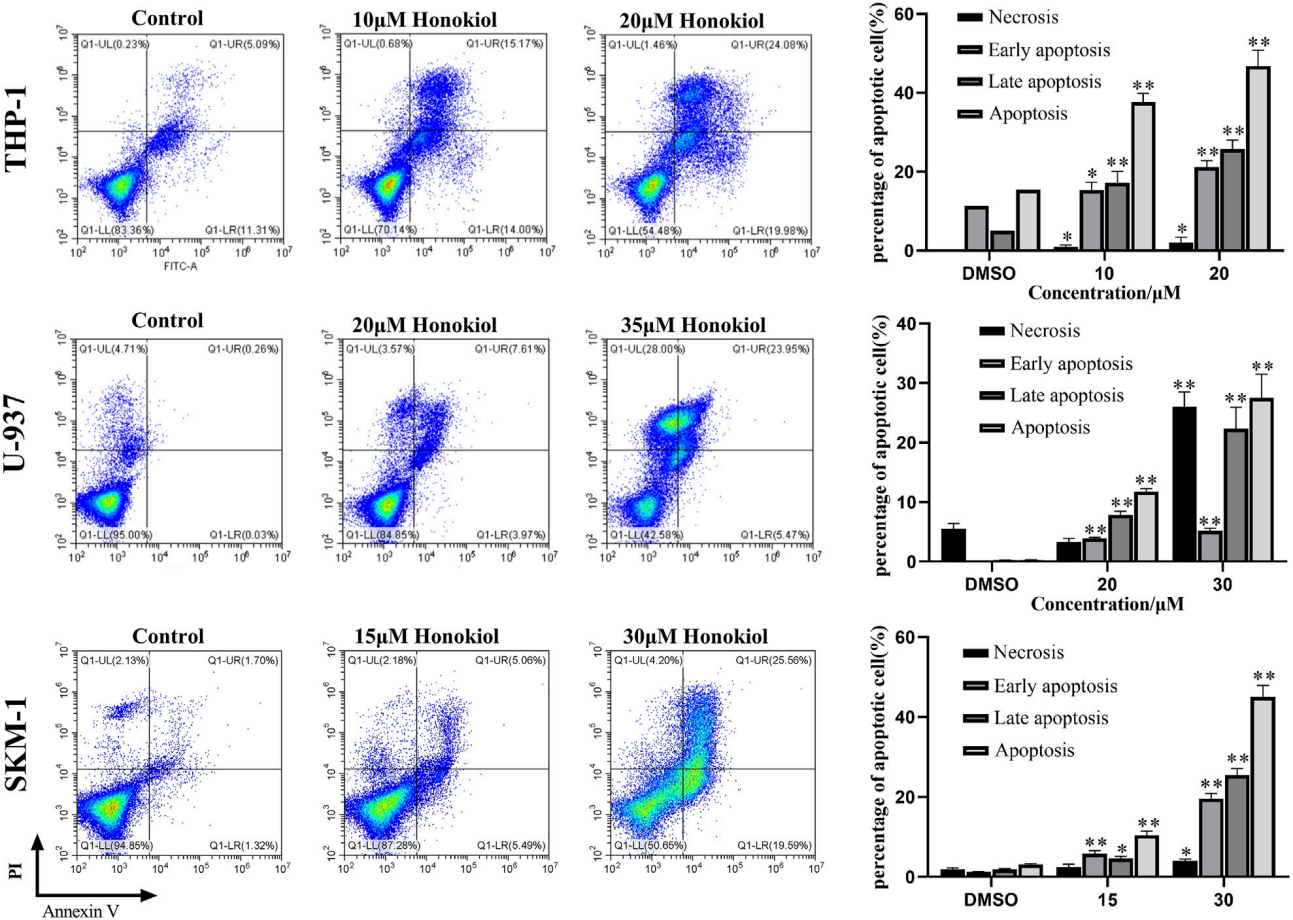
Effects of honokiol on the apoptosis of THP-1, U-937, and SKM-1 cells. The cells were incubated with the indicated concentration of honokiol for 24 h, and Annexin-V-FITC/PI staining and flow cytometry analysis were performed. The experiments were performed in triplicate. Statistical analysis of cell apoptosis was shown as bar graphs (*, *p* < 0.05, **, *p* < 0.01, compared to the control group).

To explore whether the proliferation inhibition of honokiol is related to apoptosis, we incubated THP-1, U-937, and SKM-1 cells with different concentrations of honokiol for 24 h. As shown in Figure 2, the apoptosis rate of THP-1, U-937, and SKM-1 cells rose significantly as the concentration of honokiol increased. Moreover, THP-1 was more sensitive to honokiol compared to U-937 and SKM-1. For U-937 cells, the apoptotic cells occurred mainly at the late phase. In contrast, the apoptotic cells occurred mainly at the early and late phases in THP-1 and SKM-1 cells. The results reveal that the cell death mechanism induced by honokiol varies per cell line.

### Honokiol Induced G0/G1 Phase Arrest of THP-1, U-937, and SKM-1 Cells

**FIGURE 3:**
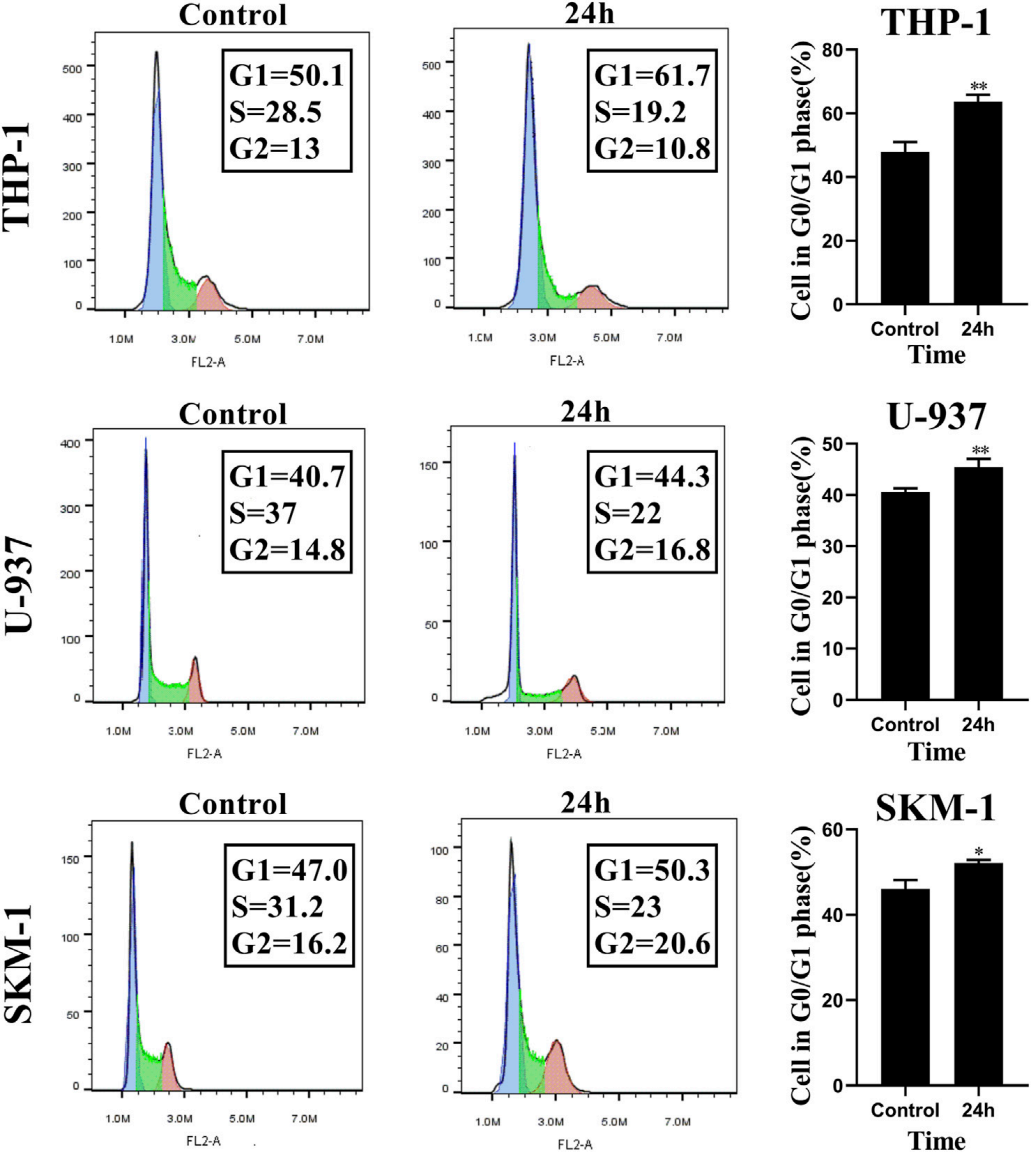
Honokiol arrests cell cycle of THP-1, U-937 and SKM-1 cells at G0/G1 Phases. The cells were incubated with the specified concentration of honokiol (THP-1: 20 μM; U-937: 35 μM; SKM-1: 30 μM) and then analyzed using flow cytometry with PI staining. Statistical analysis of G0/G1 phase cells was shown as a bar graph (*, *p* < 0.05, **, *p* < 0.01, compared to the control group). These data are from three separate replicates.

To evaluate the influence of honokiol on the cell cycle in THP-1, U-937, and SKM-1 cells, we treated these cell lines, each with a specific concentration of honokiol established by ID50 values for 24 h (THP-1: 20 μM; U-937: 35 μM; SKM-1: 30 μM). The results demonstrated that the percentage of AML cells in G0/G1 phase was significantly increased after honokiol treatment (Figure 3). These findings suggest that honokiol suppresses the proliferation of AML cells by inducing AML cell-cycle arrest at G0/G1.

### Honokiol inhibited the Leukemia Colony Formation of THP-1 and SKM-1 Cells

**FIGURE 4:**
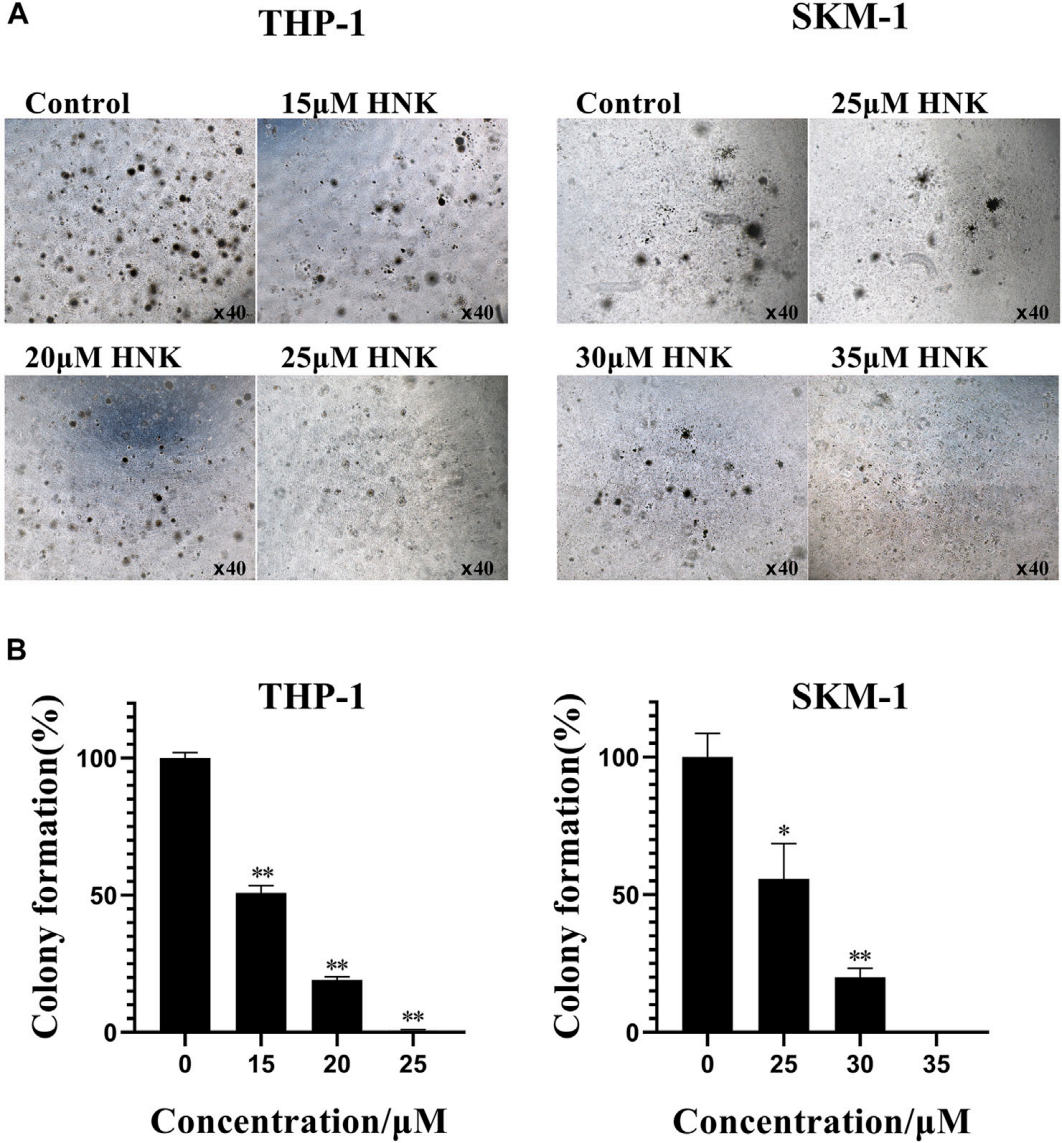
Honokiol inhibits colony formation of THP-1 and SKM-1 cells. **(A)** Cell colony results of THP-1 and SKM-1 cells. Cells were incubated with different concentrations of honokiol for 14 days and then observed under a light microscope. **(B)** Bar graphs show the statistical analysis of colony formation, presented as (*, *p* < 0.01; **, *p* < 0.001). These results represent three separate experiments.

Next, we analyzed the effect of honokiol on the colony formation of THP-1, U-937, and SKM-1 cells. As seen from Figure 4, the number of THP-1 and SKM-1 cell colonies decreased significantly as honokiol concentration increased. Eventually, neither cell line could form colonies at a high enough honokiol concentration (THP-1:25 μM; SKM-1: 35 μM). Furthermore, the U-937 cell line, whether treated with honokiol or not, could not form colonies in MethoCult™ H4100 with or without 10% 5,637 cell conditioning medium. From the results above, we conclude that honokiol can effectively inhibit the colony-formation ability of AML cells. To determine any toxicity of honokiol towards normal cells, normal hematopoietic stem/progenitor cells isolated from cord blood of healthy donors were used for colony formation assay. The result showed there were no significant differences in colony formation numbers among these groups treated with or without honokiol, indicating honokiol had no obvious toxicity for normal hematopoietic stem/progenitor cells at the concentration of less than 45 μM (Supplementary Figure S1).

### Honokiol Activated Ferroptosis Pathway in AML Cells

**FIGURE 5:**
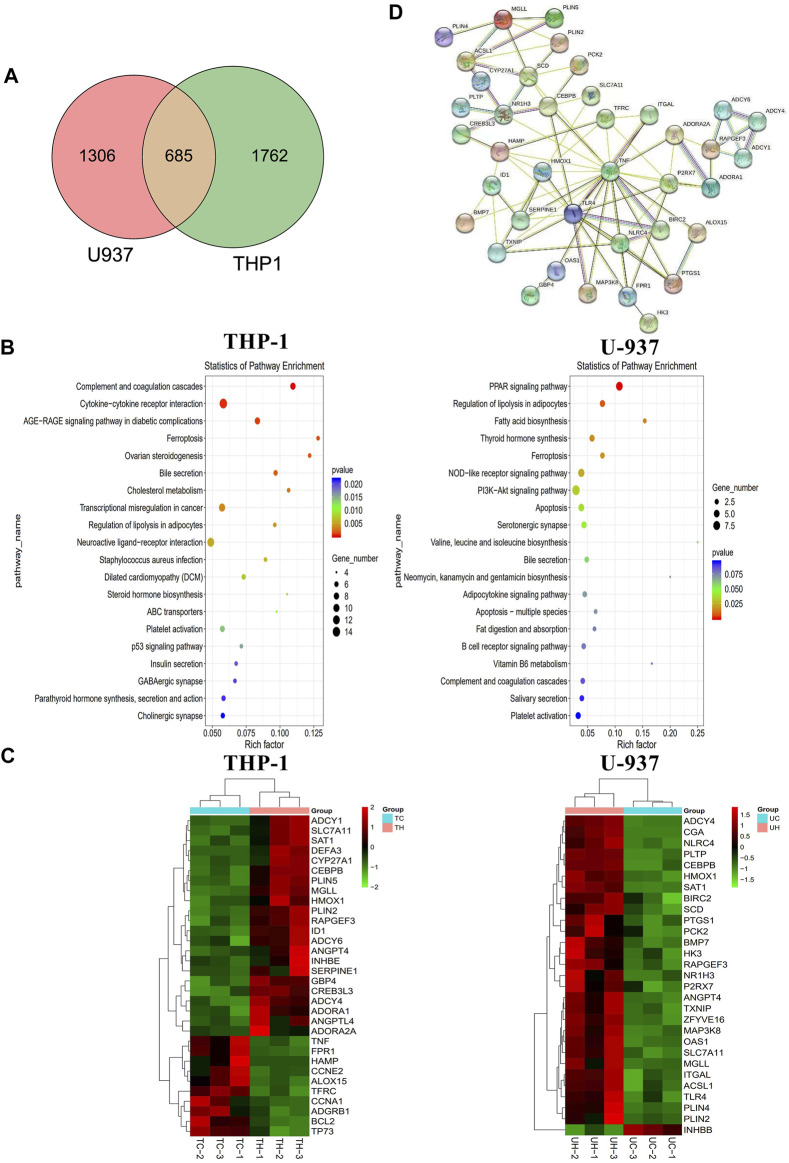
The mechanism of the inhibitory effect of honokiol on THP-1 and U-937 cells. **(A)** Venn diagram. **(B)** KEGG pathway analyses of all differentially expressed genes. **(C)** Heatmap of the partial differentially expressed genes. **(D)** Protein-Protein interaction (PPI) Network diagram.

We performed transcriptome sequencing (mRNA sequencing) in THP-1 and U-937 cells treated with honokiol in order to explore the regulation mechanism of honokiol killing leukemia cells (Supplementary Table S1). As shown in Figure 5A, there were 2,447 and 1991 differentially expressed genes in THP-1 and U-937 cells treated with honokiol, respectively. In addition, we further screened 685 genes that are common to both cell lines within this pool. These data revealed that the genes regulated by honokiol varied per cell line. The Kyoto Encyclopedia of Genes and Genomes (KEGG) analysis was performed for these differentially expressed genes in both cell lines. The top 20 signaling pathways were shown in Figure 5B, and there were four statistically significant common pathways: ferroptosis, regulation of lipolysis in adipocytes, PPAR signaling pathway, and bile secretion. Among them, ferroptosis is closely correlated with cell death induced by honokiol.

Additionally, we generated a heat map portraying the expression of the relevant genes in these pathways (Figure 5C). In the ferroptosis pathway, HMOX1 (Heme Oxygenase 1, HO-1) and SAT1 (Spermidine/Spermine N1-Acetyltransferase 1) were upregulated significantly in the group treated with honokiol, as was SLC7A11 (Solute Carrier Family seven Member 1, XCT), compared to the control group treated with DMSO only. Also, the expression of CEBPB (CCAAT Enhancer Binding Protein Beta), a gene closely related to the cell differentiation of AML (Guerzoni et al., 2006; Novikova et al., 2021), increased significantly after honokiol treatment.

In order to determine the relationships and interactions amongst these common genes in both cell lines, we performed PPI (protein-protein interaction network) analysis using STRING software. As shown in Figure 5D, HMOX1, the key member of the noncanonical ferroptosis mechanism, interacted with seven genes: SLC7A11, TFRC, HAMP, TNF, TLR4, SERPINE1, and TXNIP. Two of which, SLC7A11 and TFRC, were involved in ferroptosis.

We further detected more ferroptosis-related markers including TFR1 (TFRC), ACSL4, PTGS2, CHAC1 and SAT1 using qPCR. Three cell lines THP-1, U-937 and SKM-1 were treated with honokiol at the concentration of 20, 35, 30 μM respectively for 12h, and mRNA expression of the ferroptosis-related genes was detected by qPCR. The results showed there was no identical change for TFR1 (TFRC), ACSL4, PTGS2 and CHAC1 in these 3 cell lines, however, the mRNA expression of SAT1 increased in all the 3 cell lines treated with honokiol, which was consistent with the results of mRNA sequencing. Thus, our result suggested that SAT1 may play an important role in ferroptosis induced by honokiol (Supplementary Figure S2).

### Honokiol Enhanced Lipid Peroxidation in THP-1, U-937, and SKM-1 Cells

**FIGURE 6:**
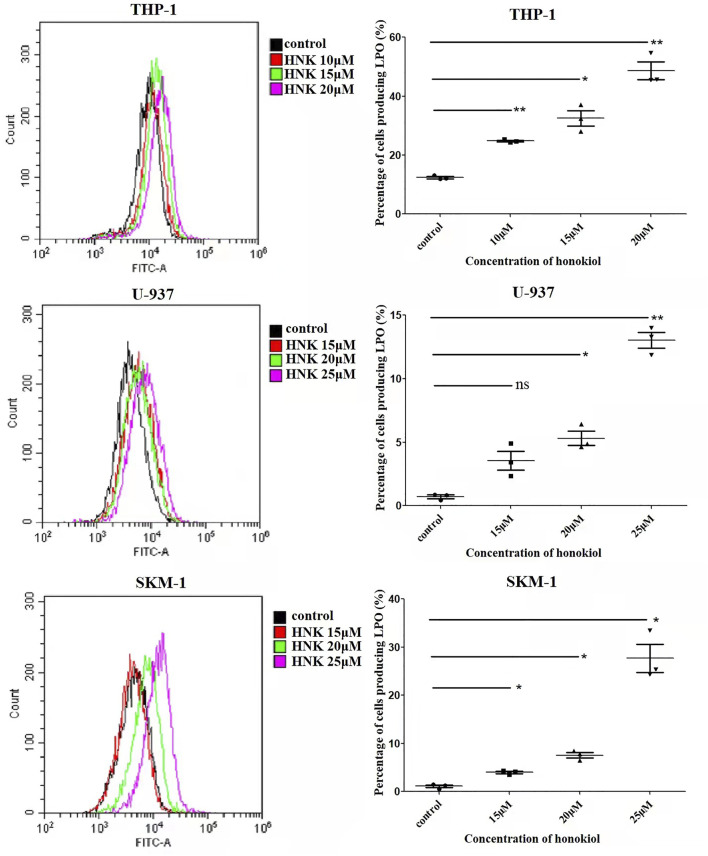
Effects of honokiol on lipid peroxidation in THP-1, U-937, and SKM-1 cells. The cells were incubated with different concentrations of honokiol for 24h, then liperfluo was added for flow cytometry analysis. Statistical analysis of lipid peroxide was presented as scatter plots (*, *p* < 0.05; **, *p* < 0.01).

As the existence of lipid peroxide serves as one of the most reliable pieces of evidence of ferroptosis, we opted to investigate the level of lipid peroxide in THP-1, U-937, and SKM-1 cells after being treated with honokiol for 12 h. It was shown in Figure 6 that honokiol elevated the expression of lipid peroxide in these cell lines. Moreover, the lipid peroxide level positively correlated with the concentration of honokiol within a specific concentration range. These data provide strong evidence for ferroptosis being induced by honokiol.

### Honokiol Induced Ferroptosis by Upregulating HMOX-1, Not by Downregulating SLC7A11

**FIGURE 7:**
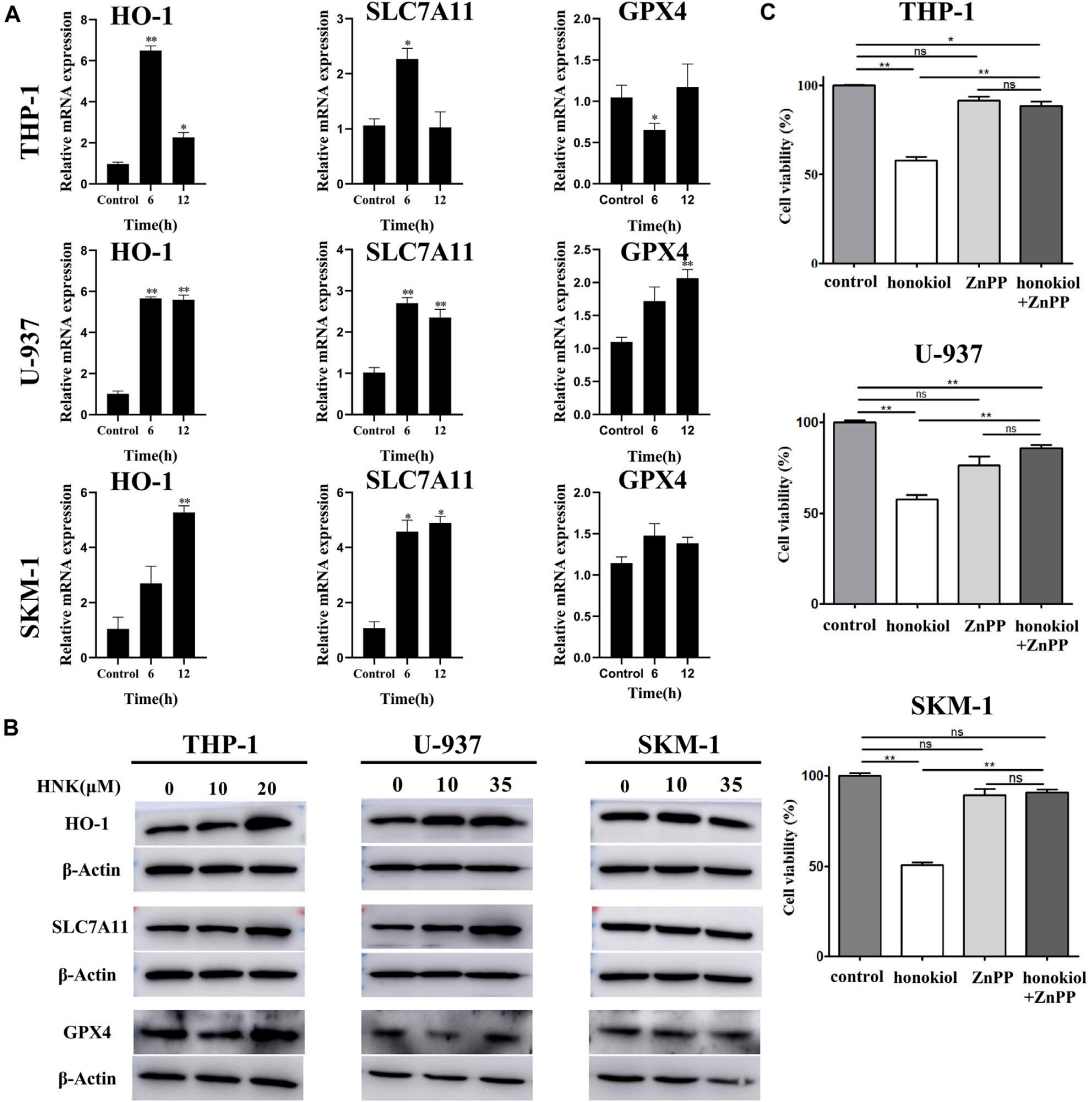
HMOX1 is a necessary molecule to induce ferroptsis in AML cells induced by honokiol. **(A)** The mRNA expression of HMOX-1, SLC7A11, and GPX4 after honokiol treatment, showing Honokiol increased the expression of HO-1 and SLC7A11. The statistical analysis of mRNA expression was presented as bar graphs (*, *p* < 0.05; **, *p* < 0.01). **(B)** Western blot results on the protein expression of HMOX-1, SLC7A11, and GPX4 in 3 cell lines. **(C)** HMOX1 inhibitor ZnPP rescued the ferroptosis of AML cells induced by honokiol. Compared with honokiol treatment group, the cell viability of honokiol combined with ZnPP treated group was significantly increased (**, *p* < 0.01).

To verify the expression of ferroptosis-related genes, qPCR and WB were used. The qPCR results (Figure 7A) indicated that the mRNA level of HMOX1 and SLC7A11 in THP-1, U-937, and SKM-1 cells treated with honokiol were upregulated significantly compared to the control group at 6 and 12 h. WB results further revealed that honokiol enhanced the protein expression level of both genes (Figure 7B). As both upregulation of HMOX1 and downregulation of SLC7A11 promoted ferroptosis, we postulate that the ferroptosis induced by honokiol in these cells mediated by the overexpression of the HMOX1 protein. The effect of SLC7A11 on ferroptosis induced by honokiol needs to be clarified in future studies.

### HMOX1 Inhibitor ZnPP Rescued the Ferroptosis of AML Cells Induced by Honokiol

To learn if HMOX1 is a necessary molecule to induce ferroptsis in AML cells induced by honokiol, three AML cell lines U-937, THP-1 and SKM-1 were treated with DMSO as control, honokiol and honokiol combined with HMOX1 inhibitor ZnPP respectively. CCK-8 assay was used to measure cell viability after treatment of the cells for 24 h and the concentrations of honokiol used were for THP-1: 20 μM, U-937: 35 μM and SKM-1: 30 μM. AS shown in Figure 7C, compared with honokiol treated group, the cell viability of honokiol combined with ZnPP treated group was significantly increased (**, *p* < 0.01), suggesting that HMOX1 inhibitor ZnPP can rescue the ferroptosis of AML cells and HMOX1 is a necessary molecule to induce ferroptosis in AML cells induced by honokiol.

To confirm ferroptosis as the major cause of cell death in AML induced by honokiol, we used Ferrostatin -1 as a ferroptosis inhibitor that has proven to inhibit ferroptosis by preventing the accumulation of ROS from lipid peroxidation. THP-1, U-937 and SKM-1 cells were pretreated with Ferrostatin-1 (5 μM) for 2 h, then honokiol was added to the cell culture at specified concentration and incubated for 24 h. Cell viability of the three cell lines was measured by CCK-8 assay. The result indicated that the ferroptosis inhibitor Ferrostatin-1 partially rescued honokiol-induced cell death in THP-1, U-937 and SKM-1 cells, suggesting that these cells underwent ferroptosis after honokiol treatment (Supplementary Figure S3).

## Discussion

AML is characterized by high chemoresistance, high recurrence risk, and poor prognosis (Siegel et al., 2021). Although several recently approved pharmacologic agents expanded AML treatment options, the outcomes of chemoresistant patients, accounting for 35–45% of new cases, are still unsatisfactory with less than 30% of long-term survival rate (Song et al., 2018; Shallis et al., 2019). Till now, practitioners have resorted to using a few Chinese herb extracts, including arsenic trioxide and HHT, to treat AML (Stahl and Tallman, 2019; Zhang et al., 2019; Zhou et al., 2020) effectively,. Notwithstanding the above, new effective therapeutic strategies or drugs for AML are still in urgent need.

Honokiol has recently attracted more attention due to its potent anti-cancer activity and low cytotoxicity (Ong et al., 2019). In this study, we evaluated the anti-leukemia effect of honokiol from one Chinese herb in AML-M5 subtype cell lines THP-1, U-937, and SKM-1 with complex karyotypes, which are closely associated with poor prognosis of AML patients (Daneshbod et al., 2019; Mrózek et al., 2019). Firstly, HNK inhibited the viability of THP-1, U-937, and SKM-1 cells, but showed almost no cytotoxicity for the normal PBMCs. Further experimental results demonstrated that HNK could induce cell cycle arrest at the G0/G1 phase and inhibit the leukemic colony formation of THP-1 and SKM-1 cells. Additionally, the dead cells mainly accumulated in the upper right quadrant with the late apoptotic cells regardless of the concentration of honokiol used. These results suggest that HNK inhibits AML cells’ proliferation by nonapoptotic programmed death manner. Our transcriptome sequencing results showed that the only cell death mode induced by honokiol in THP-1 cells was ferroptosis, whereas that in U-937 cells was ferroptosis and apoptosis (Figure 5B). Moreover, ferroptosis is statistically the primary cell death mode in U-937 cells compared to apoptosis (Figure 5B). Different from many known cell death modes, Ferroptosis is a ROS-dependent type of cell death accompanied by iron accumulation and lipid peroxidation (Tang et al., 2021a). The accumulation of lipid peroxide is considered the primary feature of ferroptosis (Li et al., 2020b). It was found that increased lipid peroxide occurred in THP-1, U-937, and SKM-1 cells, suggesting that honokiol may induce ferroptosis-like cell death.

Based on the existing data, ferroptosis is mainly divided into downregulated GPX4-induced and upregulated HMOX1-induced (Chang et al., 2018; Hassannia et al., 2018; Li et al., 2020a). Honokiol elevated the expression of HMOX1 but did not inhibit the expression of GPX4. Therefore, HMOX1 is considered directly responsible for the ferroptosis induced by honokiol.

An increasing number of studies have shown that HMOX-1 plays a dual role in ferroptosis. A proper cellular level of HMOX1 plays an antioxidative function to protect cells from ROS toxicity (Ryter and Tyrrell, 2000). However, its overexpression has pro-oxidant effects to induce ferroptosis of cells, which is dependent on intracellular iron accumulation and increased ROS content upon excessive activation of HMOX1 (Chang et al., 2018). Several HO-1 inhibitors have been discovered and are widely used. ZnPP (Zinc Protoporphyrin Zn(II)-protoporphyrin IX), a heme analog, has been widely used in studying HO-1 in ferroptosis (Tang et al., 2021b). It may competitively inhibit enzymatic activity by occupying the heme-binding site of HMOX-1. In AML cell lines THP-1, U-937, and SKM-1, ZnPP inhibited honokiol-induced cell ferroptosis significantly. Here, it was concluded that honokiol materially enhanced the expression of HMOX1, which led to the ferroptosis of THP-1, U-937, and SKM-1 cells. However, our study found that after honokiol treatment, the expression of SLC7A11 was also upregulated. This detected change is different from that in previously published data. SLC7A11 serves as one of the essential regulatory factors of ferroptosis. It protects cells from ferroptosis by importing cystine for the biosynthesis of glutathione (Stockwell et al., 2017). However, there is evidence that drugs, radiation, and other stimuli can induce cancer cells to adapt to these stress conditions by increasing the expression of SLC7A11, which is often unrelated to ferroptosis (Lin et al., 2020; Koppula et al., 2021). In tumors, SLC7A11 regulates nonferroptotic cell death, cell proliferation, drug-/radio-resistance, and tumor immunity, all of which are dependent on cystine import and/or glutamate export mediated by SLC7A11 (Zhou et al., 2020). Based on these data, we conjectured that there might be two functions of overexpressed SLC7A11 induced by honokiol. First, the overexpression of SLC7A11 due to negative feedback on lipid peroxide induced by HMOX-1 could regulate redox homeostasis by importing cystine in AML cells. Secondly, SLC7A11 may also induce cell death by excessive cysteine intake, which is likely toxic to cells (Koppula et al., 2021).

In short, honokiol has a significant inhibitory effect on THP-1, U-937, and SKM-1 cells, which is achieved by honokiol through ferroptosis, a novel cell death mechanism confirmed by mRNA sequencing technology and typical marker detection. The results of our present study suggest that honokiol triggers phenotype features of ferroptosis in AML cells by increasing the expression of HMOX1. Moreover, the HMOX1 inhibitor ZnPP rescued the ferroptosis of AML cells induced by honokiol. Therefore, these data highlight that honokiol plays a vital anti-cancer role in AML through activating the ferroptosis pathway and that HO-1 is the crucial regulating molecule in this pathway. However, it is critical to note that the results of this study also suggest that the level of SLC7A11 expression detected is contradictory to that in other published studies of ferroptosis, so the function of SLC7A11 in honokiol treatment remains to be clarified. Additionally, the anti-cancer effects of honokiol were only examined on three AML cell lines. Therefore, whether honokiol can be used as a broad-spectrum anti-leukemia drug remains uncertain. Nevertheless, our study explains the potential molecular mechanism of the anti-cancer activity of honokiol and paves the ground for further studies on the topic.

## Data Availability

The datasets presented in this study can be found in online repositories. The names of the repository/repositories and accession number(s) can be found below: https://www.ncbi.nlm.nih.gov/geo; GSE200099.
