# Supplementary material for: Honokiol Induces Ferroptosis by Upregulating HMOX1 in Acute Myeloid Leukemia Cells
Source: Front Pharmacol. 2022 May 11;13:897791. doi: 10.3389/fphar.2022.897791 (PMC9132251; doi:10.3389/fphar.2022.897791)
Supplement: Supplementary file 1 [file Image2.pdf]

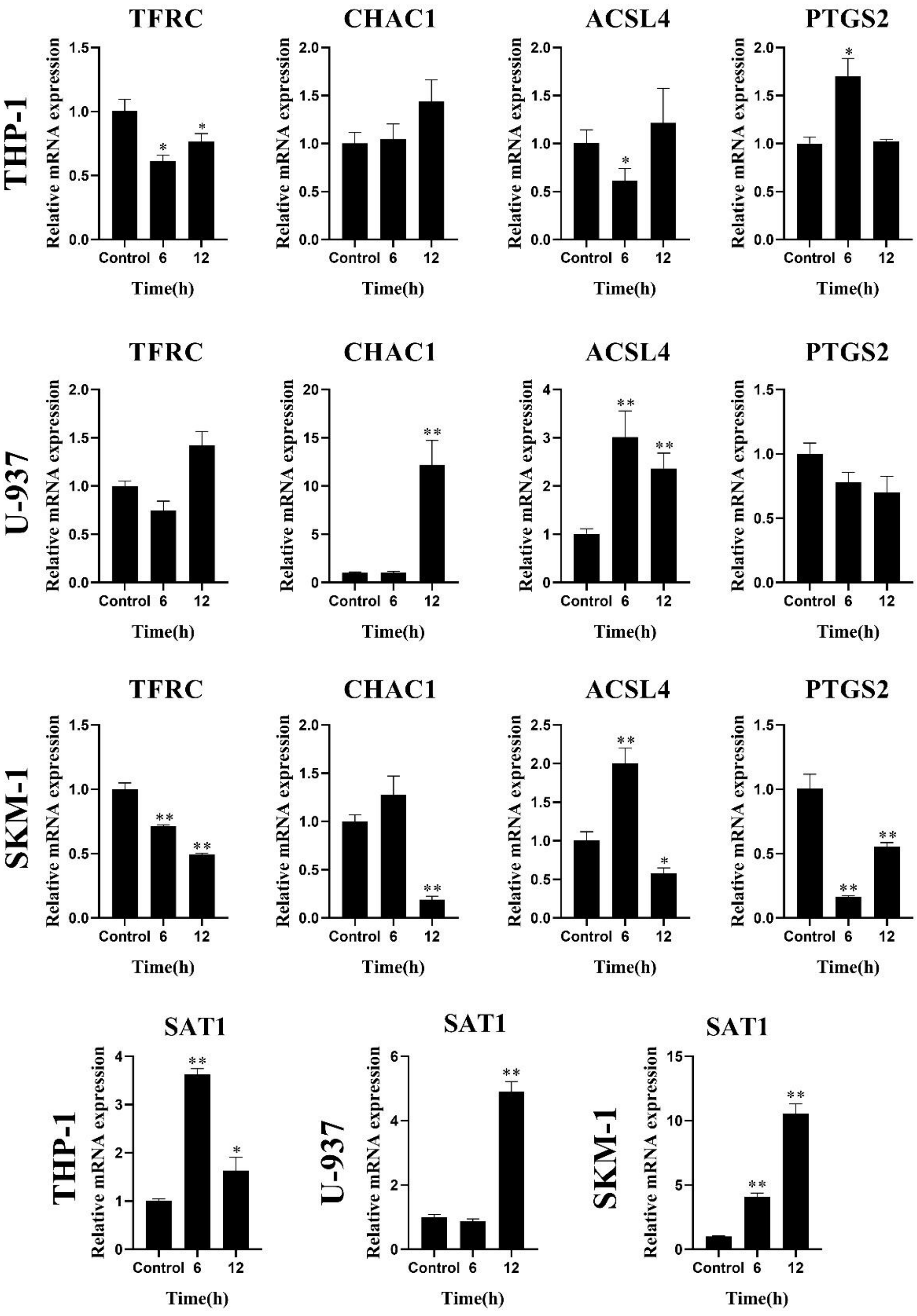

Figure S2. Determination of ferroptosis-related genes by qPCR.

Determination of ferroptosis-related markers including TFR1 (TFRC), ACSL4, PTGS2, CHAC1 and SAT1 using qPCR. THP-1, U-937 and SKM-1 cells were treated with honokiol at the concentration of 20μM, 35μM, 30μM respectively for 12h, and

mRNA expression of the ferroptosis-related genes was detected by qPCR. As shown in Figure S2, the results showed there was no identical change for TFR1 (TFRC), ACSL4, PTGS2 and CHAC1 in these three cell lines, however, the mRNA expression of SAT1 increased in all the three cell lines treated with honokiol, which was consistent with the results of mRNA sequencing. Thus, our result suggested that SAT1 may play an important role in ferroptosis induced by honokiol. Each sample was triplicated for qPCR and the bra chart represents mean±SD of relative mRNA expression of gene detected. *P<0.05, **P<0.01 by statistical analysis.
