# Supplementary material for: Honokiol Induces Ferroptosis by Upregulating HMOX1 in Acute Myeloid Leukemia Cells
Source: Front Pharmacol. 2022 May 11;13:897791. doi: 10.3389/fphar.2022.897791 (PMC9132251; doi:10.3389/fphar.2022.897791)
Supplement: Supplementary file 2 [file Image3.pdf]

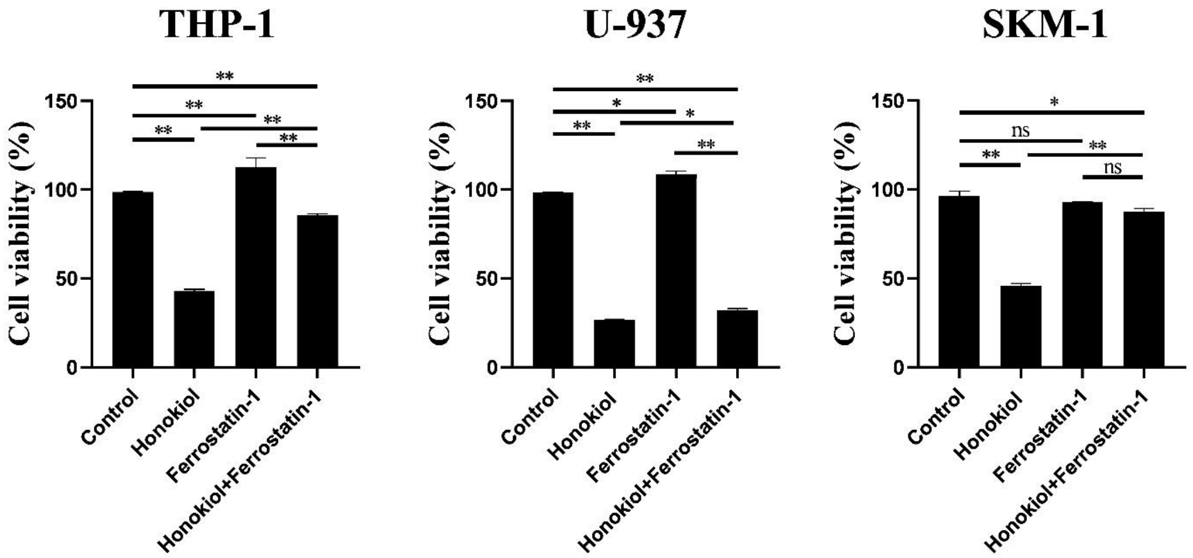

Figure S3. The effect of honokiol on cell death following ferroptosis inhibition. THP-1, U-937 and SKM-1 cells were pretreated with Ferrostatin-1 (5μM) for 2 h, then honokiol (20μM for THP-1, 35μM for U-937 and 30μM for SKM-1) was added to the cell culture and incubated for 24 h. Four hours before ending the incubation, 10 μL of CCK-8 reagent was added to each well. Four hours later, the absorbance was measured at 450 nm using a microplate reader and cell viability was calculated. As shown in Figure S3, the ferroptosis inhibitor ferrostatin-1 partially rescued honokiol-induced cell death in THP-1, U-937 and SKM-1 cells, suggesting that these cells underwent ferroptosis after honokiol treatment. Three replicated experiments were performed and the bra chart represents mean±SD of cell viability of each cell line after treatment as indicated. *P<0.05, **P<0.01 by statistical analysis.
