# Supplementary material for: Honokiol Induces Ferroptosis by Upregulating HMOX1 in Acute Myeloid Leukemia Cells
Source: Front Pharmacol. 2022 May 11;13:897791. doi: 10.3389/fphar.2022.897791 (PMC9132251; doi:10.3389/fphar.2022.897791)
Supplement: Supplementary file 3 [file Image1.pdf]

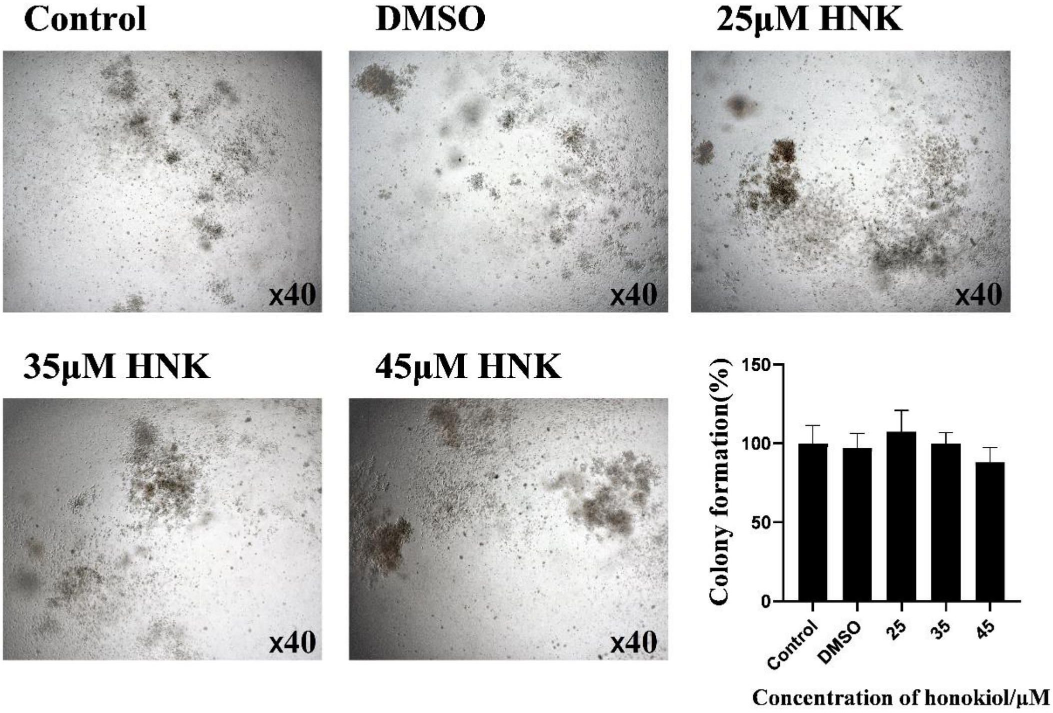

Figure S1. Determination for toxicity of honokiol toward normal hematopoietic stem/progenitor cells using colony formation assay.

Normal hematopoietic stem/progenitor cells were isolated from cord blood of health donors with informed consent. 1000 enhanced hematopoietic stem/progenitor cells were incubated with specific concentration of honokiol as indicated (25, 35 and 45μM) in 100 μl of methyl-cellulose medium per well of 96-well plate (MethoCult H4434, STEMCELL Technologies) containing cytokines and 10% FBS. The groups without honokiol treatment and with DMSO were used as controls. After about 14 days, the total colony formation numbers of containing all the BFU-E-, CFU-GM-, and CFU-GEMM- derived colonies in different groups were counted, and the statistical result was shown in the bar chart. There were no significant differences in colony formation numbers among these groups treated with or without honokiol, indicating honokiol had no obvious toxicity for normal hematopoietic stem/progenitor cells at the concentration of less than 45μM. Three replicated experiments were performed and the bar chart represents mean±SD of colony formation units.
